# Evaluating the effect of an artificial intelligence system on the anesthesia quality control during gastrointestinal endoscopy with sedation: a randomized controlled trial

**DOI:** 10.1186/s12871-022-01796-1

**Published:** 2022-10-07

**Authors:** Cheng Xu, Yijie Zhu, Lianlian Wu, Honggang Yu, Jun Liu, Fang Zhou, Qiutang Xiong, Shanshan Wang, Shanshan Cui, Xu Huang, Anning Yin, Tingting Xu, Shaoqing Lei, Zhongyuan Xia

**Affiliations:** 1 Department of Anesthesiology, Renmin Hospital of Wuhan University, 99 Zhangzhidong Road, 430060 Wuhan, Hubei Province China; 2 Department of Gastroenterology, Renmin Hospital of Wuhan University, Wuhan, China; 3 Key Laboratory of Hubei Province for Digestive System Disease, Renmin Hospital of Wuhan University, Wuhan, China; 4 Hubei Provincial Clinical Research Center for Digestive Disease Minimally Invasive Incision, Renmin Hospital of Wuhan University, Wuhan, China

**Keywords:** Endoscopy, Anesthesia, Artificial intelligence

## Abstract

**Background:**

Sedative gastrointestinal endoscopy is extensively used worldwide. An appropriate degree of sedation leads to more acceptability and satisfaction. Artificial intelligence has rapidly developed in the field of digestive endoscopy in recent years and we have constructed a mature computer-aided diagnosis (CAD) system. This system can identify the remaining parts to be examined in real-time endoscopic procedures, which may help anesthetists use anesthetics properly to keep patients in an appropriate degree of sedation.

**Aims:**

This study aimed to evaluate the effects of the CAD system on anesthesia quality control during gastrointestinal endoscopy.

**Methods:**

We recruited 154 consecutive patients at Renmin Hospital of Wuhan University, including 76 patients in the CAD group and 78 in the control group. Anesthetists in the CAD group were able to see the CAD system’s indications, while anesthetists in the control group could not. The primary outcomes included emergence time (from examination completion to spontaneous eye opening when doctors called the patients’ names), recovery time (from examination completion to achievement of the primary recovery endpoints) and patient satisfaction scores. The secondary outcomes included anesthesia induction time (from sedative administration to successful sedation), procedure time (from scope insertion to scope withdrawal), total dose of propofol, vital signs, etc. This trial was registered in the Primary Registries of the WHO Registry Network, with registration number ChiCTR2100042621.

**Results:**

Emergence time in the CAD group was significantly shorter than that in the control group (p < 0.01). The recovery time was also significantly shorter in the CAD group (p < 0.01). Patients in the CAD group were significantly more satisfied with their sedation than those in control group (p < 0.01). Vital signs were stable during the examinations in both groups. Propofol doses during the examinations were comparable between the two groups.

**Conclusion:**

This CAD system possesses great potential for anesthesia quality control. It can improve patient satisfaction during endoscopic examinations with sedation.

**Trial registration:**

ChiCTR2100042621.

**Supplementary Information:**

The online version contains supplementary material available at 10.1186/s12871-022-01796-1.

## Introduction

Anesthesia services for gastrointestinal endoscopy have risen dramatically over the past few decades. Because of its remarkable effects on discomfort relief, sedative gastrointestinal endoscopy is a common method in the clinic. According to a worldwide survey of endoscopic sedation, several countries perform > 50% of endoscopic procedures under sedation [1–4].

However, while sedation occurs during endoscopic examinations, adverse events, such as hypotension, hypertension, arrhythmia and hypoxemia, may arise [5]. Long duration and deep sedation are independent risk factors for the appearance of adverse events, resulting in greater hospital costs [6–8]. Additionally, when the interval between two sedative medication administrations is too long, the possibility of injury increases [9]. Furthermore, lower skill of anesthetists and longer duration are associated with more serious adverse events, including arrhythmia, respiratory arrest and even death [7]. Therefore, it is vital to ensure appropriate sedation during endoscopic examinations [5, 10].

Artificial intelligence (AI) has developed rapidly in recent years, and has seen extensive applications from diagnosis to prognosis in the medical field [11, 12]. Deep learning (DL) applied in the digestive-endoscope field has made remarkable achievements [13]. In a previous study, based on a series of studies on AI in endoscopy, our team developed an AI system named ENDOANGEL, which can reduce the blind spot rate during gastroscopy and increase the adenoma detection rate (ADR) during colonoscopy by monitoring observed anatomical sites, operation speed and procedure time [14–17]. We previously validated its effectiveness in improving endoscopy examination in a randomized clinical trial. Interestingly, during clinical trials, anesthetists tend to rely on the AI system, and less fatigue would occur during the anesthesia process with the assistance of AI. ENDOANGEL can remind doctors at key points, such as the injection and withdrawal point, during endoscopic procedures. The anesthetists can make an injection or withdrawal decision based on the prompt. Moreover, timely communication is needed during examinations for adding or stopping anesthetics, and a real-time reminder can inform anesthetists regarding the process of examinations directly and help doctors avoid missing the best time because of distraction. Regarding this phenomenon, we hypothesized that this system’s location-reminding and timing function would influence anesthesia quality control, because anesthetists could give proper doses of anesthetics according to the system’s implications and achieve better anesthesia quality. The present study was designed and conducted to compare anesthesia quality control between patients with or without ENDOANGEL use during routine examinations.

## Materials and methods

### Study design

We performed a single-center, randomized, single-blind trial at Renmin Hospital of Wuhan University. Patients were randomized into two groups: with or without the AI system. This study aimed to detect the influence of an AI-based diagnostic system (ENDOANGEL) on the anesthetic procedures, and the quantified indicators included (1) for patients: patient satisfaction, emergence time, recovery time, vital signs, Ramsay Sedation Scale [18] (RSS), incidence of adverse events, (2) for procedures: induction time, procedure time, total does of propofol, anesthetist satisfaction score, endoscopist satisfaction score, use of additional rescue medications.

### ENDOANGEL system

**Fig. 1:**
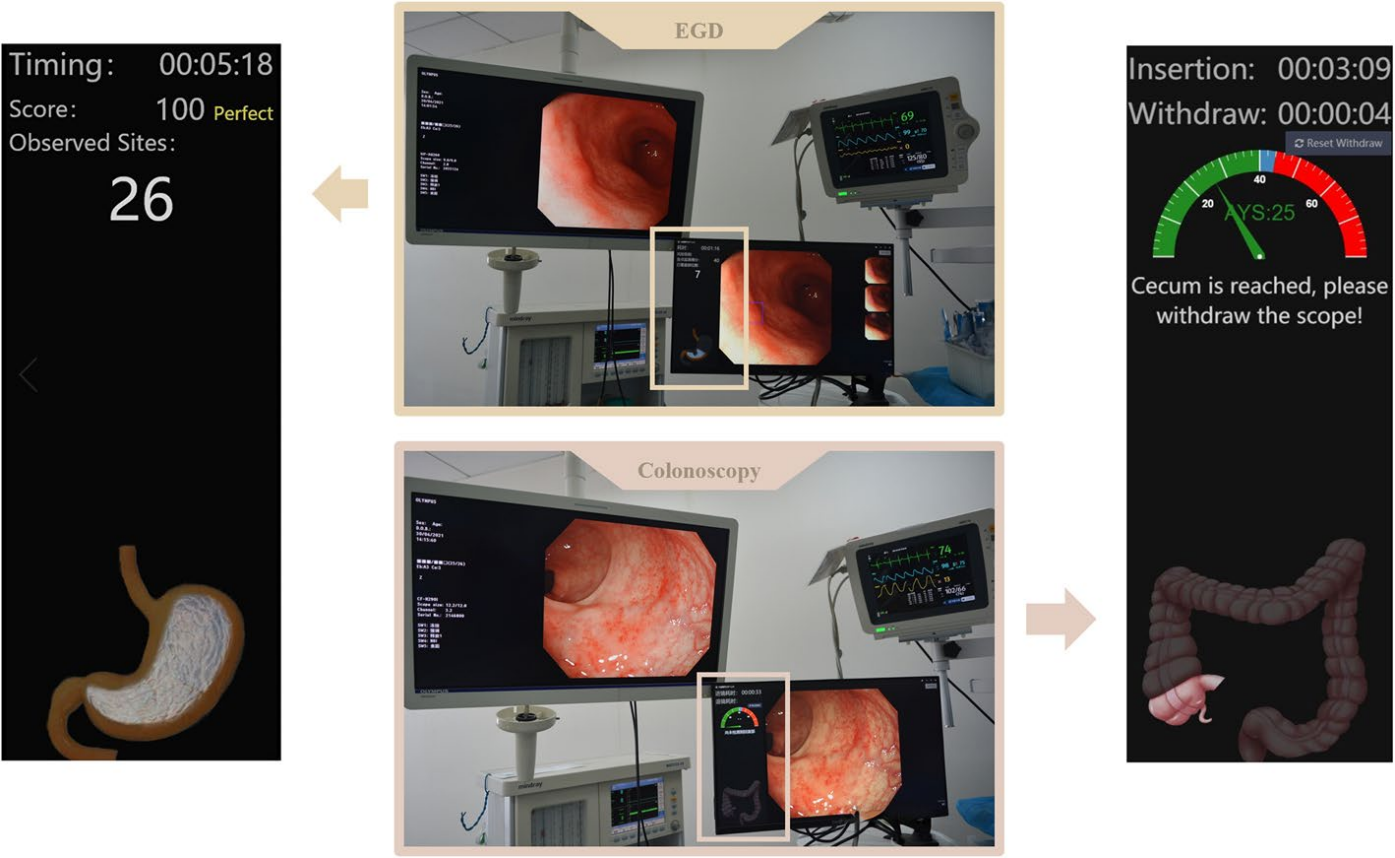
Clinical Usage of CAD system. Working scene in clinical. Clinical use of CAD system is shown in the center of this figure. Implications of this system are shown on two sides of this figure, and we can see the spending time and process of endoscopic examinations

ENDOANGEL is a deep learning-based quality control tool for digestive endoscopy that has been described in our previous studies [14–17]. Unlike ScopeGuide [19], an endoscope positioning system for colonoscopy, which can reveal the shape and position of the endoscope based on electromagnetic force. It mainly assists in leading to a successful cecum intubation to lessen the patient pain by reducing inaccurate scope movement. By comparison, ENDOANGEL is a computer-aided quality-control system working parallelly with the routine endoscopic equipment, constructed by composing of series of DCNN models. Its main functions are to monitor the visible parts of the digestive tract and record the endoscopic examination time during endoscopy. Briefly, it mainly has the following real-time functions (Fig. 1 & Supplementary Fig. 1). During gastroscopy, except for the original videos, three additional pieces of information were presented to anesthetists and endoscopists with the assistance of AI: (1) after the endoscope was inserted into the mouth, the system automatically began timing the procedure until the endoscope was drawn out; (2) the name of the anatomical landmark was observed; and (3) a virtual stomach model monitoring the anatomical sites of stomach under endoscopic examination which were unobserved.During colonoscopy, except for the original videos, three additional pieces of information were presented to anesthetists and endoscopists with the assistance of AI: (1) after the endoscope was inserted into the anus, the system automatically began timing the insertion procedure and switched to the time withdrawal procedure after the caecum was reached. (2) The withdrawal speed was monitored through a virtual dashboard on the screen; (3) When endoscope slipping occurred, a warning was presented, the system recorded the frames before scope slipping and the warning speed was persistently reported by the system until the endoscope had been reinserted to the place where the previously seen frames were detected.

### Participants

**Fig. 2:**
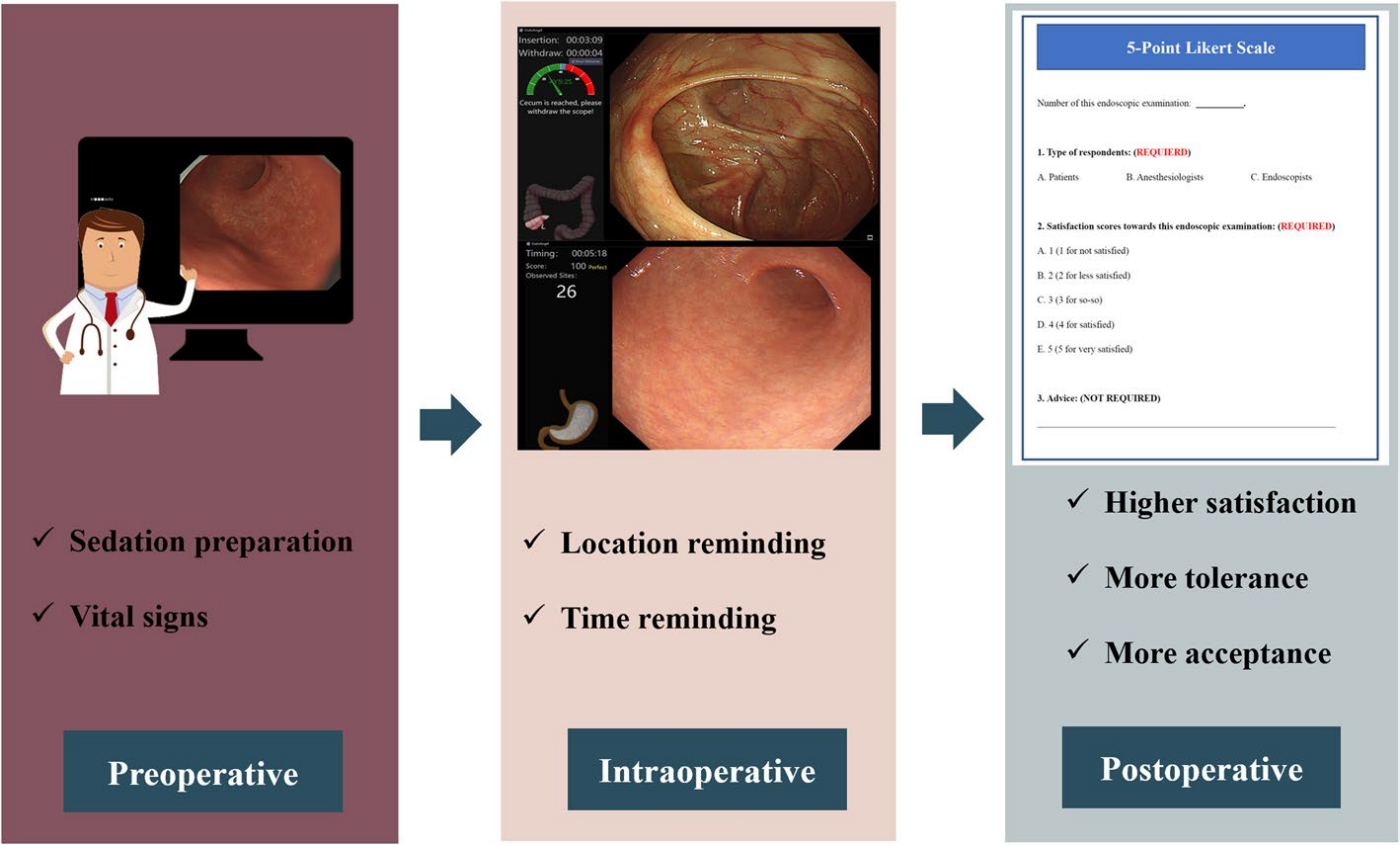
Summary chart

This trial was designed to compare anesthesia quality between patients using ENDOANGEL (computer-aided diagnosis group, CAD group) or not using ENDOANGEL (the control group) during endoscopic examinations with sedation. We recruited 154 consecutive patients aged 18 to 65 years with American Society of Anesthetists (ASA) physical status class I to III from January 25th, 2021 to March 26th, 2021. All these patients had related gastrointestinal symptoms and underwent sedative esophagogastroduodenoscopy (EGD), colonoscopy or both EGD and colonoscopy (the procedures are summarized in Fig. 2). The exclusion criteria were as follows: 1)ASA class IV or more; 2) people enrolment in other clinical trials; 3) abuse of drugs or alcohol or presence of mental disorders in the last 5 years; 4) pregnancy or breastfeeding; 5) myocardial infarction within the 6 months prior to screening; 6) uncontrolled hypertension(systolic BP of > 160 mmHg); 7) chronic lung disease; pneumonia; 8) decompensated liver or kidney disease; 9) epilepsy; 10) anticipated difficult airway; 11)asthma; 12) sedative medication allergy; 13) active upper gastrointestinal bleeding, gastric retention, pyloric obstruction or emergency gastroscopy and 14)lack of written consent.

All the sedative gastrointestinal endoscopy procedures were performed by the same experienced anesthetists and gastrointestinal endoscopists, and their total operating time was equal in both the CAD group and control group. Two researchers were responsible for intraoperative and postoperative records, and another investigator independently reviewed the data collection forms to verify data accuracy.

### Randomization and masking

Enrolled patients were randomly allocated into a CAD group or a control group at ratio of 1:1. Randomization was performed in blocks of 4 in a 1:1 ratio and was computer-generated by a random allocation sequence. Anesthetists in charge of anesthetics administration were aware of the allocation. All of the patients ,as well as the other investigators who recorded the postoperative results, were blinded to the allocation.

### Procedures

All patients were given a 4 L/min oxygen supplementation via nasopharynx tube and received standard monitoring, including heart rate (HR), electrocardiogram (EGD), SpO_2_, noninvasive blood pressure (NIBP), and respiratory rate (RR). Rehydration was given using Sodium Acetate Ringer’s Injection at 8ml/Kg/h. The same initial sedative administration plan was used in all enrolled patients. Sufentanil was given at 0.05 µg/kg within one minute [20, 21], and then propofol (normally 1.5-3 mg/kg) was injected until the depth of sedation was suitable for endoscopic procedures. The degree of sedation was assessed by using the RSS (details are shown at Supplementary methods and materials), and a Ramsay Score between 2 and 4 indicated an appropriate sedation level. If the RSS score was below 2 or a sudden body movement occurred, propofol was injected at 1/3 of the induction dose.

Vital signs and RSS scores were recorded at six time points, including at baseline (T0), after anesthesia induction (T1), at the beginning of endoscope insertion (T2), three minutes after insertion (T3), at the end of the examination (T4), 5 min after entering the recovery room (T5) and the time leaving the recovery room (T6).The induction time, endoscopy time, emergence time and recovery time were also recorded.

The following times were defined as clinical experience :induction time was defined as the time from sedative administration to successful sedation (from the beginning of propofol administration to disappearance of the eyelash reflex in patients). Procedure time was the time from endoscope insertion to the end of the examination (from scope insertion to scope withdrawal). Emergence time represented the time from examination completion to eye opening when doctors called the patients’ names. Recovery time was the time from examination completion to orientation, at which point patients could state their names.

Intraoperative adverse events (e.g. cough, hiccup, moving, hypertension, arrhythmia, hypoxemia and hypotension) and postoperative adverse events (e.g. dizziness, nausea, respiratory depression, dysphoria and hypoxemia) were recorded, as well as the extra use of medication during adverse events.

The satisfaction of the patients, endoscopists and anesthetists with the procedure [22] was recorded by a 5-point Likert scale [23–25] (1 for not satisfied, 2 for less satisfied, 3 for neutral, 4 for satisfied and 5 for very satisfied). High scores indicated a high degree of satisfaction. Anesthetist satisfaction was defined as the overall satisfaction with the sedation portion of endoscopy [22]. Satisfaction scores were completed by both endoscopists and anesthetists at the end of the procedure, and by patients before their discharge from the recovery room. In Fig. 2, we summarize the simple procedures and the expected results.

### Outcomes

Our primary endpoints included: emergence time, recovery time and patient satisfaction scores. Secondary endpoints included anesthesia induction time, procedure time, total does of propofol, vital signs [including mean arterial pressure (MAP), SpO2, HR, RR], ratio of RSS at 2 to 4 at T4, T5 and T6, number of patients corresponding to each RSS score at each time point, and satisfaction scores of the anesthetists and endoscopists. The incidence of adverse events and the use of additional rescue medications were used for safety evaluation.

### Equipment

All examinations in this trial were performed on gastrointestinal scopes with white-light imaging. [(EG-590ZW, EG-590WR, EG-L590ZW; Fujifilm, Tokyo, Japan), (GIF-H290, GIF-HQ290, GIF-Q260, GIF-XQ260, GIF-H260Z; Olympus Medical Systems, Co., Ltd., Tokyo, Japan)]

### Statistical analysis

We calculated the sample size by comparing two means in the two groups. To acquire the maximum sample size, we provided 80% power or more to decrease the emergence time from 240 s to 180 s with a variance of 130 s. We obtained these data based on a pretest at Renmin Hospital of Wuhan University. The sample size at 74 per group was calculated with a two-sided significance level. We considered the drop-out rates of this study to be 10%. The final sample size in need was 164. We used a two-group chi-squared test for categorical variables, and a two-sample t test for continuous variables. The Mixed Model for Repeated Measurements (MMRM) was applied to detect the change in vital signs at different times and to analyze the effects of groups on vital signs, assuming that repeated measurement data had equal correlations. The model uses vital signs at different moments after baseline as the dependent variable, group as the major factor and baseline vital signs as the covariant and includes a random intercept to fit the correlation of repeated measurement data to analyze group effects, time effects and interaction effects of time and group. The threshold of significance was a *p* value over 0.05. The sample size calculation was acquired at http://powerandsamplesize.com. Statistical analysis was performed using IBM SPSS Statistics version 26 and R version 4.0.2.

### Ethics

Ethical approval for this study (EA-19-003) was provided by the Ethical Committee of Renmin Hospital of Wuhan University, Wuhan, Hubei Province, China (Chairperson Prof H. Chen) on 21 January 2021. Written informed consent was obtained from each patient before examinations. This trial was registered at the Primary Registries of the WHO Registry Network and the registration number is ChiCTR2100042621. The first registration was on 24/01/2021.

## Results

### Demographics and characteristics

**Fig. 3:**
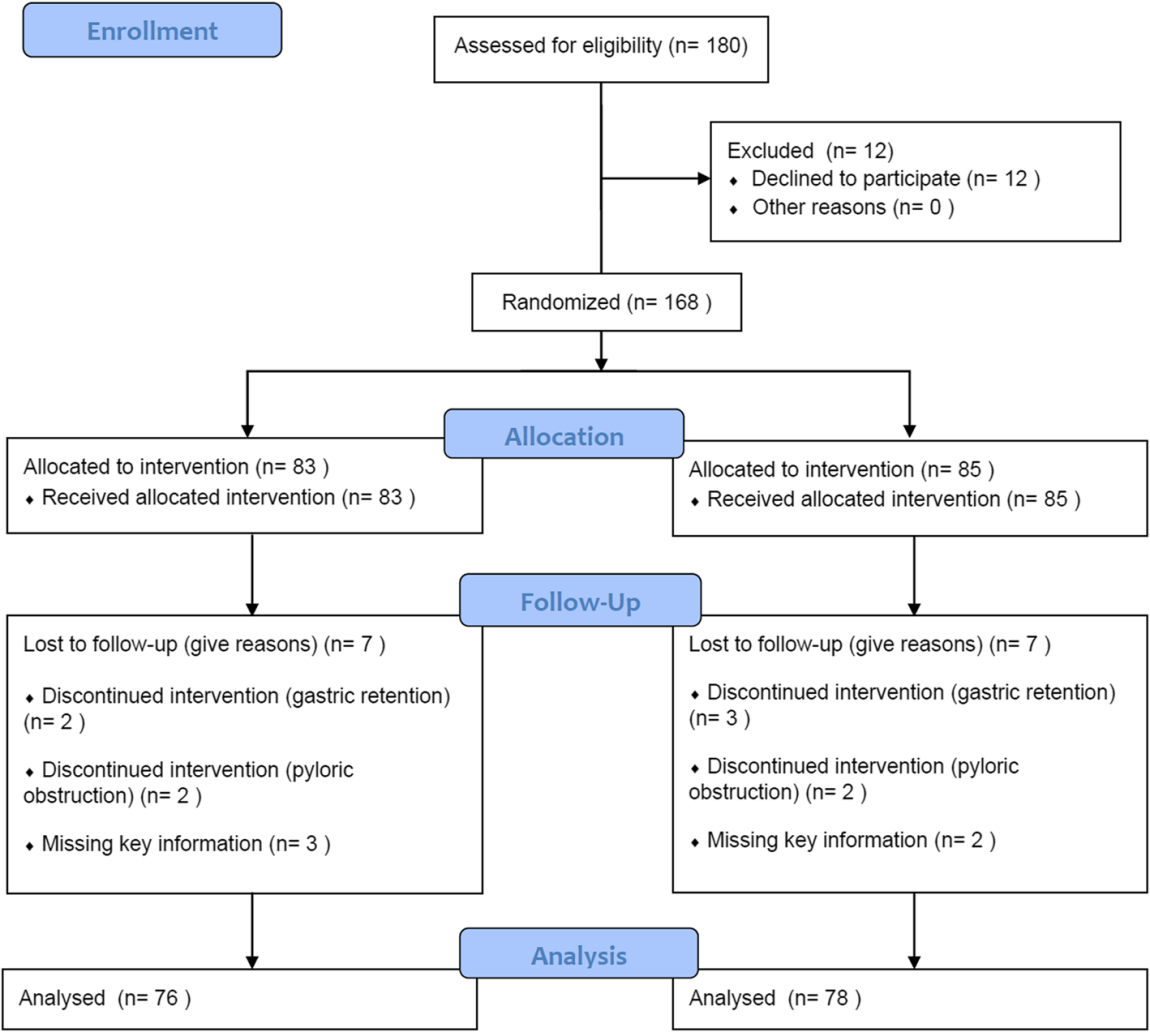
Flow diagram

Figure 3 shows the flow diagram in this study. We enrolled a total of 180 consecutive patients from January 25th, 2021 to March 26th, 2021 at Renmin Hospital of Wuhan University. After excluding 12 patients who eventually declined to be enrolled, 9 patients who did not complete endoscopy due to gastric retention or pyloric obstruction, and 5 patients who were missing key information, we included 76 patients in the CAD group and 78 patients in control group in final analysis. The baseline information of these patients is shown in Table 1. There was no significant difference in age, sex, BMI, ASA classification or number of different kinds of endoscopies, as well as comorbidities, including hypertension, diabetes, ischemic heart disease, cerebral vascular insufficiency, arrhythmia and COPD, between these two groups. Endoscopic diagnosis was similar between the two groups.

**Table 1:** Baseline of patients’ characteristics. Data is presented as Mean ± SD (standard deviation, SD) or numbers

Characteristics	CAD Group (*n* = 76)	The Control Group (*n* = 78)	*p* value
**EGD/colonoscopy/EGD + colonoscopy**	24/16/36	26/18/34	0.892
**Age, year (SD)**	45.99 ± 12.79	47.82 ± 13.04	0.380
**Gender, n (%)**			
Male	35 (46.05)	35 (44.87)	0.883
**BMI, kg/m2 (SD)**	22.96 ± 3.17	22.61 ± 3.14	0.502
**ASA (I/II/III)**	59/16/1	63/13/2	0.685
**Recruitment, n (%)**			
Outpatient	68 (89.47)	67 (85.90)	0.500
**Indication, n (%)**			
Screening/ Symptomatic/ Surveillance	10/46/20	12/45/21	0.909
**Comorbidities, n (%)**			
Hypertension	9 (11.84)	10 (12.82)	0.854
Diabetes	2 (2.63)	1 (1.28)	0.982
Ischemic heart disease	0 (0.00)	4 (5.13)	0.135
Cerebral vascular insuffificiency	2 (2.63)	2 (2.56)	1.000
Arrhythmia	0 (0.00)	0 (0.00)	
COPD	3 (3.95)	1 (1.28)	0.594
**Intraoperative Biopsy, n (%)**	25 (32.89)	30 (38.46)	0.471
^a^ **Endoscopic diagnosis**			
Reflux Esophagitis	4	5	-
Barrett Esophagus	3	4	-
Chronic Superficial Gastritis	23	19	-
Chronic Atrophic Gastritis	14	15	-
Gastric Polyp	10	8	-
Peptic Ulcer	4	6	-
Colorectal Polyp	15	13	-
Colorectal Erosion	4	5	-
Other Dieases	19	14	-

^a^One patient can have diverse endoscopic diagnosis

### Primary outcomes

The primary outcomes are shown in Table 2. The emergence time of patients in the CAD group was, significantly shorter than that in the control group [( 2.73 ± 2.16 )vs. (4.73 ± 3.34), (*p* < 0.01)]. The recovery time of the CAD group was also significantly shorter than that in the control group [(4.01 ± 2.40 min) vs. (6.55 ± 3.80 min, (*p* < 0.01)]. Patient satisfaction scores were significantly higher in the CAD group comparing with the control group [(4.54 ± 0.50) vs. (4.04 ± 0.49), (*p* < 0.01)].

**Table 2:** Outcomes of examinations. Data is presented as Mean ± SD (standard deviation, SD) or numbers

Patients’ parameters	CAD Group (*n* = 76)	the Control Group (*n* = 78)	*p* value
**Induction time, min (Mean ± SD)**	1.02 ± 0.31	1.05 ± 0.30	0.551
**Procedure time, min (Mean ± SD)**			
Procedure time (EGD), min (Mean ± SD)	4.55 ± 1.15	4.12 ± 0.95	0.165
Procedure time (colonoscopy), min (Mean ± SD)	15.02 ± 8.18	15.42 ± 5.71	0.872
Procedure time (EGD + colonoscopy), min (Mean ± SD)	22.46 ± 6.89	22.24 ± 9.76	0.914
**Emergence time, min (Mean ± SD)**	2.73 ± 2.16	4.73 ± 3.34	^**^0.000
**Recovery time, min (Mean ± SD)**	4.01 ± 2.40	6.55 ± 3.80	^**^0.000
**Propofol dose, mg (Mean ± SD)**	190.07 ± 50.15	205.58 ± 58.08	0.080
**Ramsay, n (%)**			
^ a^Ramsay1, n (%)	50 (65.79)	33 (42.31)	^**^0.003
^ a^Ramsay2, n (%)	75 (98.68)	65 (83.33)	^**^0.001
^ a^Ramsay3, n (%)	76 (100.00)	77 (98.72)	0.242
^b^ **Satisfaction**			
Patient Satisfaction (Mean ± SD)	4.54 ± 0.50	4.04 ± 0.49	^**^0.000
Anesthetist Satisfaction (Mean ± SD)	4.21 ± 0.61	3.55 ± 0.67	^**^0.000
Endoscopist Satisfaction (Mean ± SD)	4.24 ± 0.53	3.79 ± 0.54	^**^0.000

^a^ Numbers of RSS scores between 2 to 4. Ramsay1 for sedation scoring immediately after procedure, Ramsay2 for sedation scoring 5 min after entering recovery room and Ramsay3 for sedation scoring when leaving the recovery room. ^b^ Quantization of satisfaction was based on 5-point Likert scales. **p value < 0.01. EGD, esophagogastroduodenoscopy

### Secondary outcomes

**Fig. 4:**
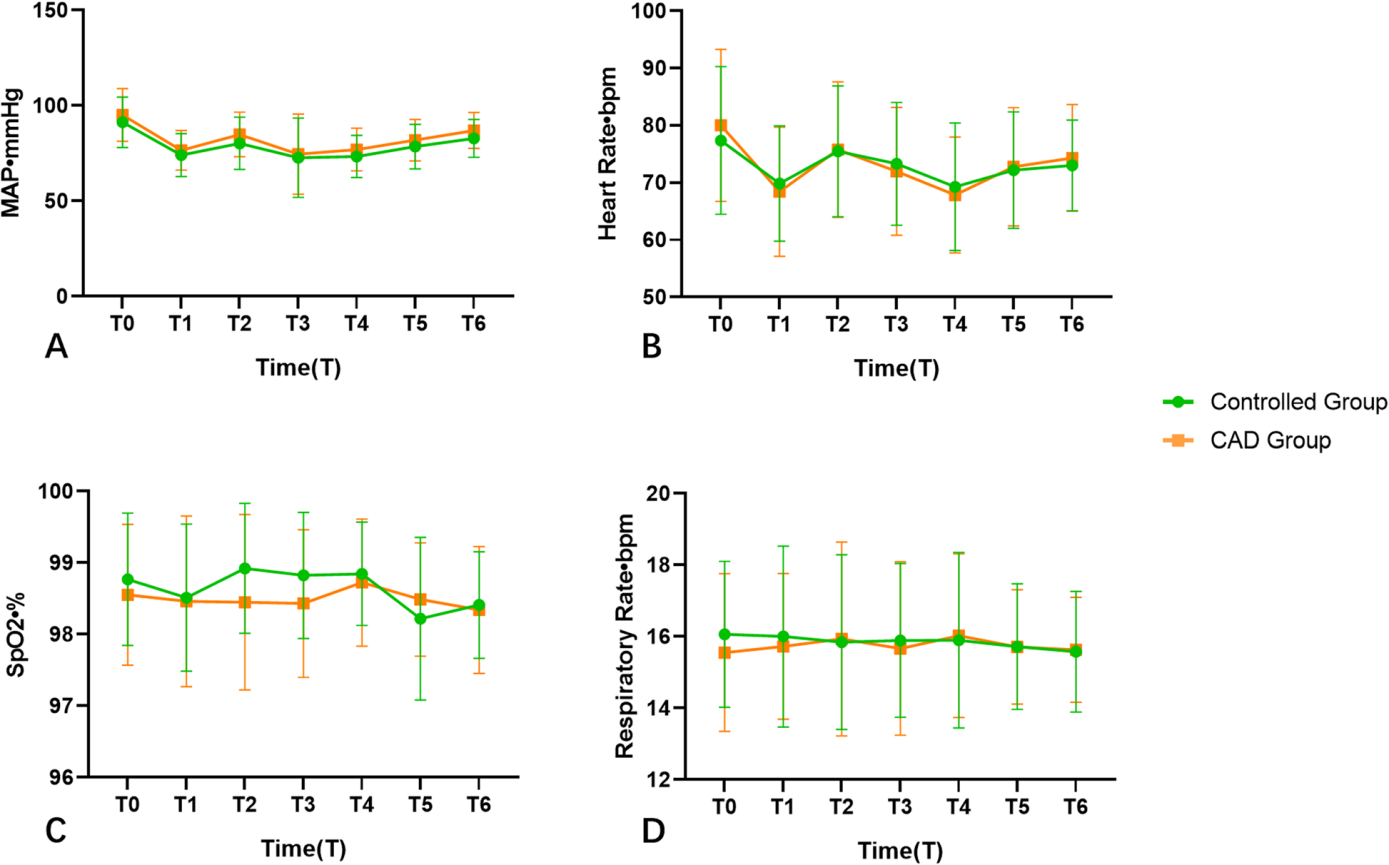
Vital signs at six moments of two groups. **A** Comparison MAP between two groups. **B** Comparison HR between two groups. **C** Comparison SpO_2_ between two groups. **D** Comparison RR between two groups. *p value < 0.05. MAP, mean arterial pressure. HR, heart rate. RR, respiratory rate

As shown in Table 2, the induction time and procedure time were not significantly different between the CAD group and the control group. The total usage of propofol was slightly but not significantly decreased in the CAD group compared with the control group. However, compared to that in the control group, the ratio of patients at RSS scores of 2–4 is significantly higher in the CAD group when the procedures were finished (*p* < 0.01) and five minutes after entering the recovery room (*p* < 0.01). The ratio of patients at RSS scores of 4 in the CAD group is lower than that of the control group when the procedures finished (*p* = 0.026). After five minutes entering the recovery room, the ratio of patients at RSS scores of 2 is higher and the ratio of scoring 3 is lower in the CAD group comparing with that in the control group (*p* < 0.01). (Supplementary Table 2). Anesthetist satisfaction scores (4.21 ± 0.61 in the CAD group and 3.55 ± 0.67 in the control group, *p* < 0.01) and endoscopist satisfaction scores (4,24 ± 0.53 in the CAD group and 3.79 ± 0.54 in the control group, *p* < 0.01) are higher in the CAD group than the control group. There was no between-group difference in MAP, HR or RR (*p* > 0.05). There are time-group interaction effects on SpO_2 _(Fig. 4 & Supplementary Table 1).

### Incidence of adverse events

As shown in Table 3, the common adverse events during the sedative endoscopy procedure were choking, cough, hiccup, moving, arrhythmia, hypoxemia and hypotension. Compared to the control group, the incidence of total adverse events was much lower [14 (18.42%) in the CAD group vs.27 (34.62%) in the control group, *p* < 0.05], and patients were less likely to report cough [4 (5.26%) in the CAD group vs.14 (17.95%) in the control group, *p* < 0.05]. In contrast, the incidence of postoperative adverse events [1 in the CAD group (hypoxemia) and 2 in the control group (1 for nausea and 1 for hypoxemia)] and the use of other medications [2 in the CAD group (1 for phenylephrine and 1 for methoxamine hydrochloride) and 3 in the control group (1 for phenylephrine, 1 for atropine and 1 for dopamine)] were not significantly different between these two groups.

**Table 3:** Safety analysis. Adverse events and use of other medications (n). Data is expressed as numbers, (%) of patients

	CAD Group (*n* = 76)	The Control Group (*n* = 78)	*p* value
**Intraoperative adverse events, n (%)**	**14 (18.42)**	**27 (34.62)**	^*^ **0.023**
Chocking cough	4 (5.26)	14 (17.95)	^*^0.014
Hiccup	5 (6.58)	5 (6.41)	1.000
Moving	1 (1.32)	5 (6.41)	0.224
Hypertension	0	0	-
Arrhythmia	2 (2.63)	1 (1.28)	0.982
Hypoxemia	1 (1.32)	1 (1.28)	1.000
Hypotension	1 (1.32)	1 (1.28)	1.000
**Postoperative adverse events, n (%)**	**1 (1.32)**	**2 (2.56)**	**1.000**
Dizziness	0	0	-
Nausea	0	1 (1.28)	1.000
Respiratory depression	0	0	-
Dysphoria	0	0	-
Hypoxemia	1 (1.32)	1 (1.28)	1.000
**Use of other medications, n (%)**	**2 (2.63)**	**3 (3.84)**	**1.000**
Phenylephrine	1 (1.32)	1(1.28)	1.000
Atropine	0	1(1.28)	1.000
Dopamine	0	1 (1.28)	1.000
Methoxamine hydrochloride	1 (1.32)	0	0.494

## Discussion

This randomized controlled trial, to our knowledge, is the first study to evaluate the effects of the CAD system (named ENDOANGEL) on anesthesia quality control during gastrointestinal endoscopy with sedation. We found that patients in the CAD group had shorter emergence time and recovery time along with higher scores of patient satisfaction. Furthermore, the incidence of overall adverse events was lower in the CAD group.

Apropriate sedation can benefit patients undergoing endoscopic procedures [26]. Propofol has a short half-life and is a satisfactory intravenous anesthetic. However, patients can easily enter deep sedation due to its narrow therapeutic window. Opioids, dexmedetomidine, and ketamine have been used in combination with propofol for endoscopic sedation, which may help to maintain stable hemodynamics and minimize the risks for respiratory depression and other adverse events. Sufentanil, a potent synthetic opioid agent, has been used in combination with other medications for outpatient sedation [20, 21]. Zhang L, et al. reported that a loading dose of sufentanil 0.05 µg/kg combined with propofol appeared to be a good choice in patients undergoing gastrointestinal endoscopic procedures [27]. In the current study, we also applied 0.05 µg/kg of sufentanil to decrease the adverse events caused by a large dose of opioid analgesic drugs and reduced the use of propofol at the same time to achieve adequate sedation. Remarkably, there was no significant difference of propofol dosage between two groups, this follows from two reasons: (1) This system is not a specialized detection system for monitoring the depth of sedation and giving feedback in real time, so it cannot calculate precise dose of propofol. (2) Propofol administration was conducted by two comparable experienced attending anesthetists, and their extensive propofol-administration experience avoided certain dosing mistakes, but it is worth mentioning that patients in the CAD groups have shorter recovery time and fewer adverse events because their propofol administration time points, with the assistance of ENDOANGEL’s real-time functions, were more precise than the corresponding time points in the control group.

AI is trending in the field of medicine, and it provides a better-personalized and more popular health care system.It is widely used in anesthesia. Christopher R et al. [28] demonstrated a machine-learning-based remote perioperative patient risk assessment system, but this system needs experts to provide remote suggestions combined with real-time information. Two studies [29, 30] reported the development of computer-aided personalized sedation systems assist in administering propofol automatically, but these are auto-propofol-administration machines with overdosing risks. We believe a real-time propofol-administration-reminder system will be safer than the system mentioned above, because the sedative medication dose is under flexible control. ENDOANGEL can prompt regarding unchecked parts, endoscopists’ scope-insertion and scope-withdrawal times, which reminds anesthetists to give a supplementary dose of anesthetics at a proper time and ensures that patients stay at a moderate-sedation status. Moderate sedation can facilitate a better medical examination than deep sedation for patients and reduce adverse events  [5, 10]. We believe that patients are more willing to undergo sedation with ENDOANGEL assistance during endoscopic examinations based on their satisfaction scores.

This is the first study to detect whether the DL-based system ENDOANGEL influences anesthesia quality control. We found that the system had a positive effect on anesthetists during endoscopic examinations. Anesthetists using ENDOANGEL can seize the adding or stopping points of anesthetic agents more accurately than those without this system. Patients, anesthetists and endoscopists were more satisfied with the anesthesia quality in the CAD group because of the shorter recovery time and fewer complications, and this resulted in better endoscopic procedures [14–17]. Since this system has timing and location reminder functions, it may play a role in reminding anesthetists of the time point for adding or stopping anesthetics. Considering its potential in quality control, we can add some functions to this system, targeting sedative depth supervision and feedback, which makes it more visual and intelligent for anesthetic administration.

There are still several limitations of this study. First, this was a single center clinical trial. A larger clinical trial is being planned to validate the effectiveness of this system in anesthesia procedures to improve its broad applicability. Second, this was not a double-blind trial. Because anesthetists needed real-time information from this system, the randomization was unblinded to them from the beginning of each endoscopic examination, and this may have weakened the proving strength of both the endoscopists and anesthetists’ satisfaction assessment. However, the patients’ satisfaction scores were convincing because they were blinded to the randomization. To avoid selection bias, the postoperative observer was blinded to the randomization, and we had an investigator supervise their recording. Third, we only used a 5-point Likert scale to evaluate the participants’ satisfaction because it is simple, intelligible and manageable. The AI system cannot change the pain intensity on the patients, but it can improve patients’ satisfaction scores according to shortening emergence time & recovery time and reducing adverse events. Fourth, endoscopists and anesthetists had rare communications during the endoscopic examinations, but we did not record the numbers of conversations between them during the process. While communications between endoscopists and anesthetists were allowed during the endoscopic procedures, it was difficult to standardize these communications, which were very subjective. It is difficult to obtain real-time feedback from endoscopists during the procedures unless anesthetists actively ask for it, which may lead to missing the time window. ENDOANGEL, as an intelligent system, can monitor unobserved landmarks, time the procedure and provide real-time and more standardized information to anesthetists.

ENDOANGEL is a comprehensive, AI-assisted diagnostic system. It can provide anesthetists with real-time indications, and these clinicians can then make a proper decision regarding anesthetic administration. ENDOANGEL can improve anesthesia quality control during endoscopic examinations with sedation.

## Supplementary Information

**Additional file 1.**

## Data Availability

The data supporting the findings of this study are not publicly available because of institutional policy but are available from the corresponding author on reasonable request.

## Abbreviations

CAD: computer-aided diagnosis
AI: Artificial intelligence
ADR: adenoma detection rate
ASA: American Society of Anesthesiologists
EGD: esophagogastroduodenoscopy
HR: heart rate
EGD: electrocardiogram
NIBP: noninvasive blood pressure
RR: respiratory rate
RSS: Ramsay sedation scale
MAP: mean arterial pressure
MMRM: Mixed Model for Repeated Measurements
