# Supplementary material for: Evaluating the effect of an artificial intelligence system on the anesthesia quality control during gastrointestinal endoscopy with sedation: a randomized controlled trial
Source: BMC Anesthesiol. 2022 Oct 7;22:313. doi: 10.1186/s12871-022-01796-1 (PMC9540709; doi:10.1186/s12871-022-01796-1)

**Supplementary methods and materials**

**Ramsay Sedation Scale**

| **Score** | **Level of Sedation** |
| --- | --- |
| 1 | Patient is anxious and agitated or restless, or both |
| 2 | Patient is co-operative, oriented, and tranquil |
| 3 | Patient responds to commands only |
| 4 | Patient exhibits brisk response to light tactile stimuli or loud auditory stimulus |
| 5 | Patient exhibits sluggish response to light tactile stimuli or loud auditory stimulus |
| 6 | Patient exhibits no response |

**Supplementary Table1. Vital signs of patients at six moments. Data is presented as Mean±SD (standard deviation, SD)**

| **Vital Signs** | **time** | **The controlled group**  **(n=78)** | **CAD**  **group(n=76)** | ***p* value^▲^** | ***p* value^△^** | |
| --- | --- | --- | --- | --- | --- | --- |
| HR | T0 | 77.37±12.89 | 80.00±13.29 | 0.215 | | 0.183 |
|  | T1 | 69.86±10.07 | 68.42±11.28 | 0.405 | |  |
|  | T2 | 75.49±11.41 | 75.76±11.84 | 0.883 | |  |
|  | T3 | 73.30±10.72 | 71.99±11.18 | 0.471 | |  |
|  | T4 | 69.28±11.14 | 67.86±10.11 | 0.407 | |  |
|  | T5 | 72.19±10.16 | 72.76±10.33 | 0.730 | |  |
|  | T6 | 73.01±7.90 | 74.33±9.30 | 0.345 | |  |
| MAP | T0 | 91.28±13.20 | 95.12±13.87 | 0.081 | | 0.317 |
|  | T1 | 74.05±11.21 | 76.59±10.37 | 0.146 | |  |
|  | T2 | 80.21±13.68 | 84.84±11.68 | ^*^0.025 | |  |
|  | T3 | 72.72±20.72 | 74.55±20.96 | 0.586 | |  |
|  | T4 | 73.33±11.07 | 76.95±11.19 | ^*^0.046 | |  |
|  | T5 | 78.53±11.68 | 81.91±10.81 | 0.064 | |  |
|  | T6 | 82.77±9.86 | 86.99±9.46 | ^**^0.008 | |  |
| RR | T0 | 16.06±2.04 | 15.55±2.21 | 0.137 | | 0.767 |
|  | T1 | 16.00±2.53 | 15.72±2.04 | 0.457 | |  |
|  | T2 | 15.85±2.44 | 15.93±2.71 | 0.833 | |  |
|  | T3 | 15.89±2.15 | 15.67±2.42 | 0.553 | |  |
|  | T4 | 15.9±2.45 | 16.03±2.29 | 0.737 | |  |
|  | T5 | 15.72±1.76 | 15.71±1.60 | 0.978 | |  |
|  | T6 | 15.58±1.69 | 15.63±1.47 | 0.831 | |  |
| SpO_2_ | T0 | 98.77±0.92 | 98.55±0.99 | 0.161 | | ^*^0.009 |
|  | T1 | 98.51±1.03 | 98.46±1.19 | 0.771 | |  |
|  | T2 | 98.92±0.91 | 98.45±1.23 | ^**^0.007 | |  |
|  | T3 | 98.82±0.88 | 98.43±1.03 | ^*^0.014 | |  |
|  | T4 | 98.85±0.72 | 98.72±0.89 | 0.349 | |  |
|  | T5 | 98.22±1.14 | 98.49±0.79 | 0.091 | |  |
|  | T6 | 98.41±0.75 | 98.34±0.89 | 0.606 | |  |

▲. Compare vital signs between two groups at certain time. △. Detect whether time-group interaction effects exist. * *p* value < 0.05. ** *p* value < 0.01. MAP, mean arterial pressure. HR, heart rate. RR, respiratory rate.

**Supplementary Table2: number of patients corresponding to each RSS score at each time point (n). Data is expressed as RSS 2/RSS 3/RSS 4.**

|  | **The controlled group**  **(n=78)**  **^△^RSS2/RSS3/RSS4** | **CAD group**  **(n=76)**  **RSS2/RSS3/RSS4** | ***p* value** |
| --- | --- | --- | --- |
| T1 | 0/2/76 | 0/4/72 | 0.328 |
| T2 | 2/22/50 | 3/21/51 | 1.000 |
| T3 | 8/10/57 | 6/10/58 | 0.883 |
| T4 | 5/6/22^a^ | 14/18/18^b^ | ^*^0.026 |
| T5 | 38^a^/22^a^/5 | 61^b^/13^b^/1 | ^*^0.005 |
| T6 | 70/6/1 | 71/5/0 | 1.000 |

**^△^**RSS2:number of people whose RSS was 2; RSS3:number of people whose RSS was 3; RSS4:number of people whose RSS was 4;******p* value< 0.05;^a,b^ The number of different ratings of RSS score at 2、3、4 between the two groups at the same time point is different .

**Supplenmentary Figure1. Function sketch map of ENDOANGEL system**


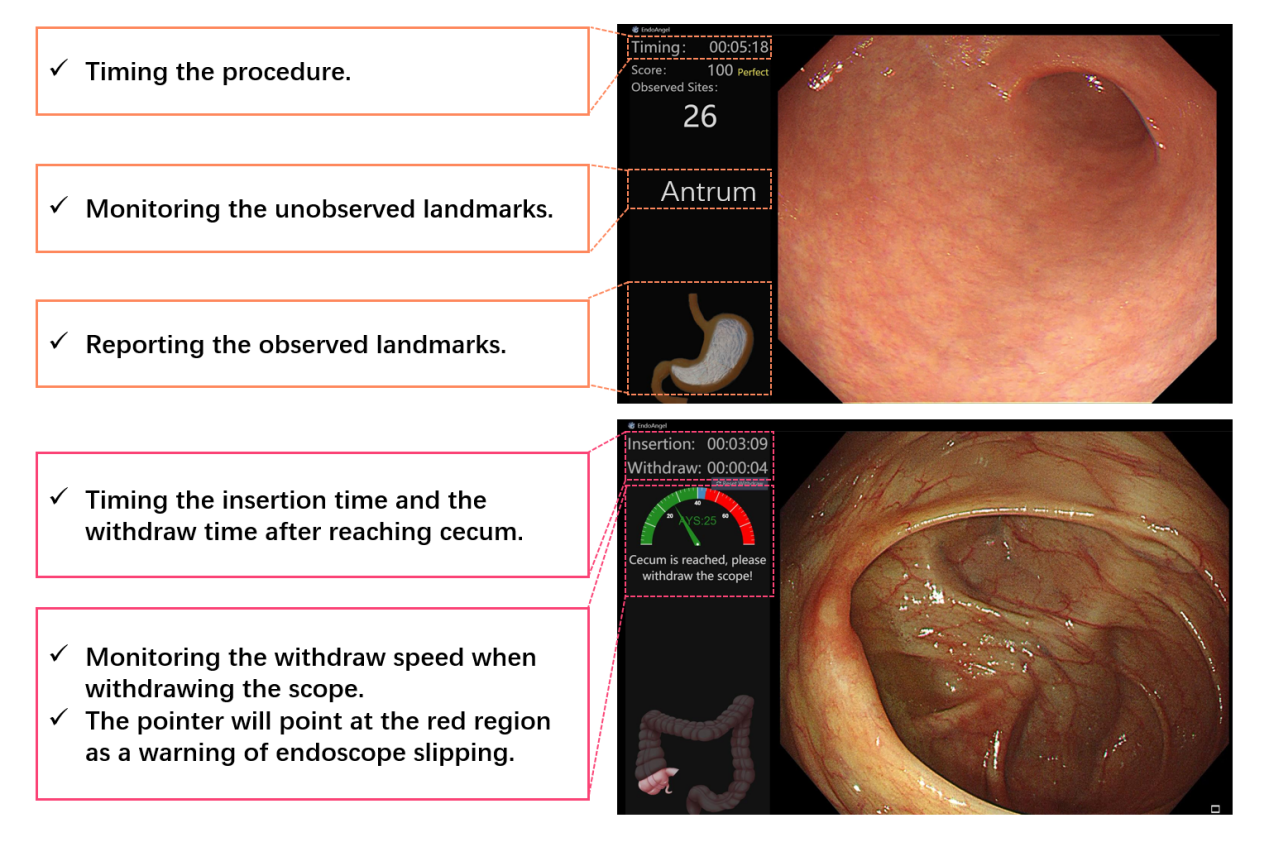

Supplement: Supplementary file 1 — Additional file 1. [file 12871_2022_1796_MOESM1_ESM.docx]
